# Supplementary material for: Impact of contaminant size and density on their incorporation into sea ice
Source: Nat Commun. 2025 May 12;16:4375. doi: 10.1038/s41467-025-59608-2 (PMC12069638; doi:10.1038/s41467-025-59608-2)
Supplement: Supplementary file 1 — Supplementary Information [file 41467_2025_59608_MOESM1_ESM.pdf]

Supplementary Information for Impact of contaminant size and density on their incorporation into sea ice

Alice Pradel[1*], Rudolf Hufenus[2], Martin Schneebeli[3] and Denise M. Mitrano[1*]

[1] Institute of Biogeochemistry and Pollutant Dynamics (IBP), Department of Environmental Systems Science, ETH Zürich, Switzerland
[2] Empa, Swiss Federal Laboratories for Materials Science and Technology, Laboratory of Advanced Fibers, St. Gallen, Switzerland
[3] WSL Institute for Snow and Avalanche Research SLF, Davos, Switzerland
[*] Corresponding authors

Table of Contents:

Tables

Table S1: Properties of the model contaminants used……………………………………………p.2
Table S2: Width of brine channels…………………………………………………………………p.4
Table S3: Mass balance of species from the columns after experiments………………………..p.10
Table S4: : p-values of Welch t-test between control and contaminants for ice and brine……….p.10

Figures

Figure S1: Microscopy images of model colloidal and particulate contaminants……………………p.2
Figure S2: Mass of water (g) recovered in different ice, brine and the underlying liquid……………p.3
Figure S3: pH of the artificial seawater before freezing (Reference) and in the different compartments (Liquid, Brine and Ice) recovered after freezing……………………………………………p.3
Figure S4: Structure of three different centrifugated ice cores……………………………………..p.4
Figure S5: Concentration of different species measured in bulk ice including the top layer of ice with PP-MPs, relative to the initial concentration in the entire column……………………………………p.4
Figure S6: Hydrodynamic diameters and zeta-potentials of NPs and nanosoot after freezing……p.6
Figure S7: Sizes of NPs and Nanosoot aggregates recovered from ice observed by optical microscopy…………………………………………………………………………………………p.7
Figure S8: Evolution of the ice thickness (mm) as a function of freezing duration (hours)………...p.8
Figure S9: Mass, salinity and pH of ice for controls and treatments…………………………………p.11
Figure S10: Mass, salinity and pH of brine for controls and treatments……………………………p.12

Sections

Section S1 : Settling/Rising velocities of MPs…………………………………………………………p.5
Section S2 : Leaching of Yttrium from PP-MP in ASW…………………………………………………p.8
Section S3 : Pre-concentration of NPs by flocculation…………………………………………………p.9

Supplementary References……………………………………………………………………p.13

Table S1: Properties of the model contaminant used: Sizes with *Molecular weight; $^{+}$Hydrodynamic diameter; ^{x}Area equivalent diameter), and, when relevant to the contaminants' behavior, their densities.

Model contaminant	Type of contaminant	Size	Density
**Rose Bengal**	Dissolved	0.973 kDa*	
**Nanosoot**	Colloidal	117 ± 3 nm^{+}	
**Nanoplastic**		210 ± 19 nm^{+}	
**Small PET-MPs**	Particulate	35.6 ± 14.4 μm^{x}	1.39 g cm^{-3}
**Large PET-MPs**		90.0 ± 29.8 μm^{x}	
**Small PP-MPs**		45.7 ± 20.7 μm^{x}	0.91 g cm^{-3}
**Large PP-MPs**		72.3 ± 37.2 μm^{x}	

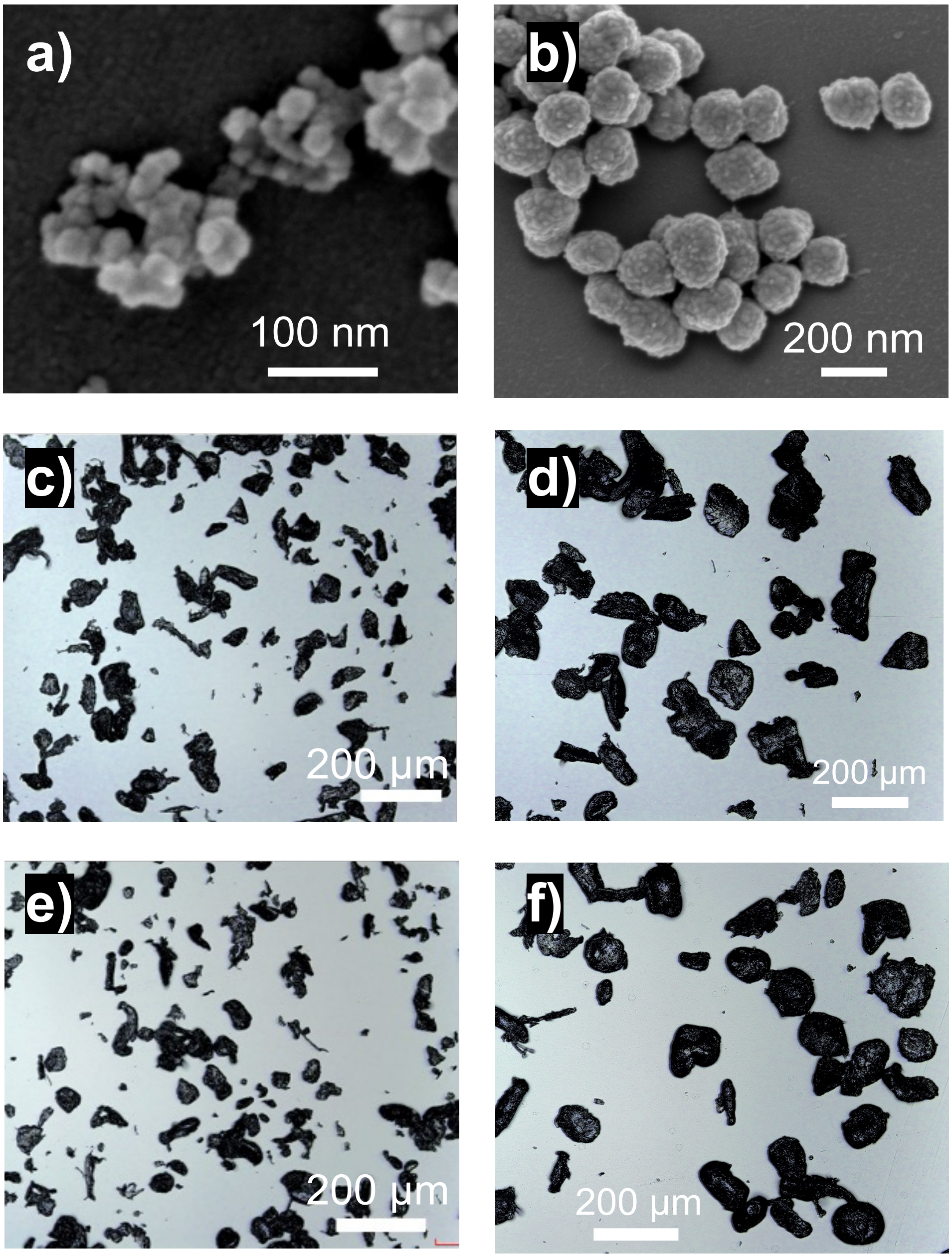

Figure S1: Scanning electron microscopy images of a) nanosoot and b) nanoplastics and optical microscopy images of c) small PP-MPs, d) large PP-MPs, e) small PET-MPs and f) large PET-MPs

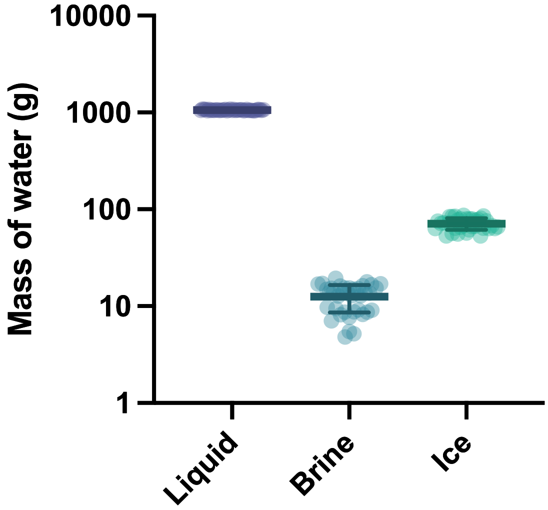

Figure S2: Mass of water (g) recovered in different compartments. All data points across all experimental variants are shown. Line represents the mean and error bars represent the standard deviation. n = 41)

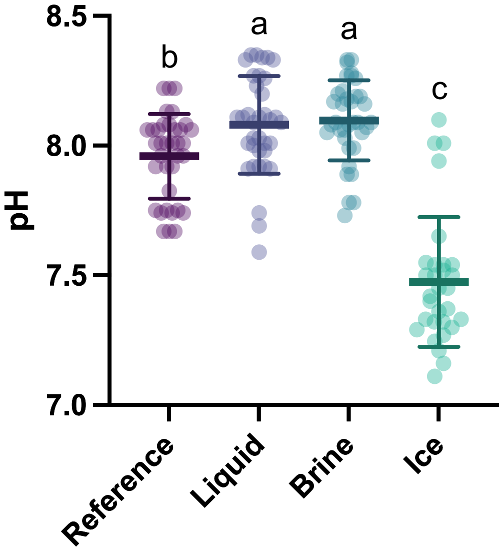

Figure S3: pH of the artificial seawater before freezing (Reference) and in the different compartiments (Liquid, Brine and Ice) recovered after freezing. All data points across all experimental variants are shown. The line represents the mean and error bars represent the standard deviation. (n = 41. p = 0.05)

The pH of the reference, liquid and ice were proportional to their salinity (linear correlation of $R^2 = 0.937$). However, the pH of the brine increased less than its salinity. This had already been observed experimentally and can be caused by a combination of processes, including freeze rejection of hydrolysable ions and gases (e.g.: HCO_3^- and CO_2) and carbonate precipitation.[1,2]

Table S2: Width of brine channels (Average ± Standard deviation. n = 51). Rectangles in Fig. S3 show a selection of main channels in green, bottom (last) growth in orange, small porosity in pink, horizontal lenses in blue, top first mms in yellow in Fig. S3

	Large porosity				Small porosity
	Main channels	Bottom (last) growth	Horizontal lenses	Top first mms	
Width (µm)	121.2 ± 30.4	127.9 ± 37.1	240.6 ± 58.4	126.2 ± 28.8	50.5 ± 14.1

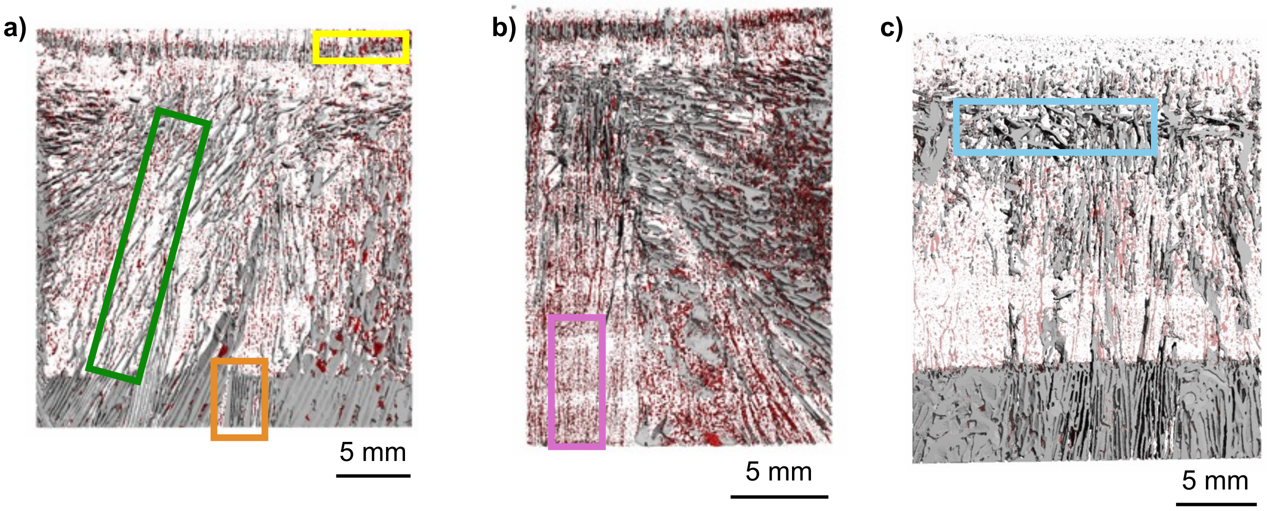

Figure S4: Structure of three different centrifugated ice cores with a) nanosoot, b) NPs and c) PP-MP. Images were obtained by micro-X-Ray tomography and show the ice (transparent), the large porosity (grey) and the small porosity containing brine (red). From left to right, rectangles show: main channels (green), bottom (i.e., last) growth (orange), top first mms (yellow), small porosity (pink), horizontal lenses (blue).

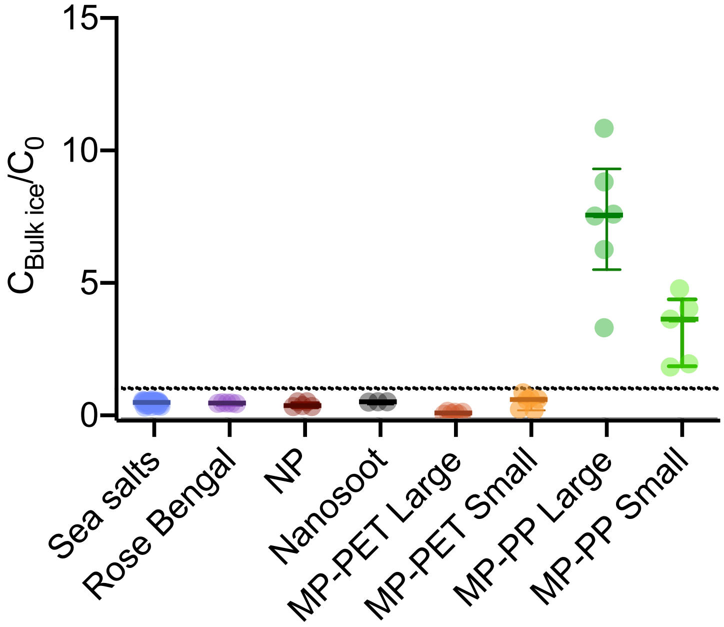

Figure S5: Concentration of different species measured in bulk ice ($C_{bulk\ ice}$) including the top layer of ice with PP-MPs, relative to the initial concentration in the entire column (C_0). Data points, median and IQR. n= 41 for Sea salts, n= 9 for PET-MPs Large, n= 6 for NP, PET-MPs Small, PP-MPs Large and Small, n = 5 for Rose Bengal, n = 3 for Nanosoot.

Supplementary Section 1 : Settling/Rising velocities of MPs

When approximating the particles to have a spherical geometry, according to Stokes law, the rising velocity was 664 mm hour^{-1} and 265 mm hour^{-1} for large and small PP-MPs, respectively. Conversely, for the large and small PET-MPs, the settling velocity was 3208 mm hour^{-1} and 501 mm hour^{-1}, respectively. Both rising and settling velocities were higher than the initial ice front advancement of approximately 10 mm hour^{-1} which suggests that higher degrees of enrichment and depletion from ice should have been observed. However, the fact that high-density MPs were still quantified in the ice and low-density MPs remained in the liquid after 19 hours could possibly be explained by three effects, either alone or in combination:

i) the rising/settling velocity of fragments has been shown to be 2 to 5 times slower than for spheres[3];

i) for each particle type there is a continuum of rising and settling velocities due to variability in the size distribution

ii) the columns were slightly agitated which could have slightly reduced both settling and rising rates compared to estimated values in still water

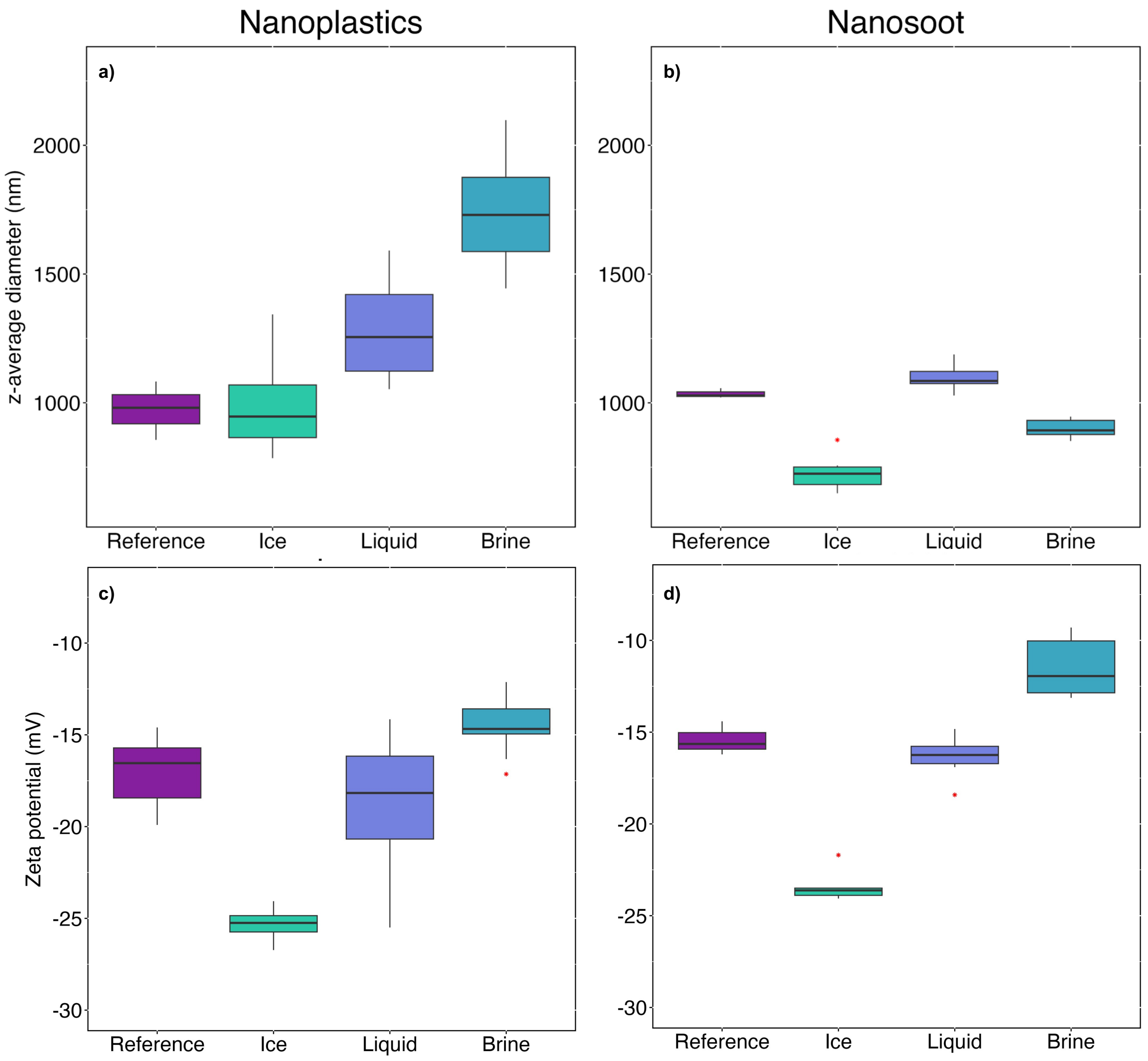

Figure S6: z-average (hydrodynamic) diameter of a) NPs and b) nanosoot and zeta-potential of c) NPs and d) nanosoot (n=3 for Reference and n=6 for Ice, Liquid and Brine)

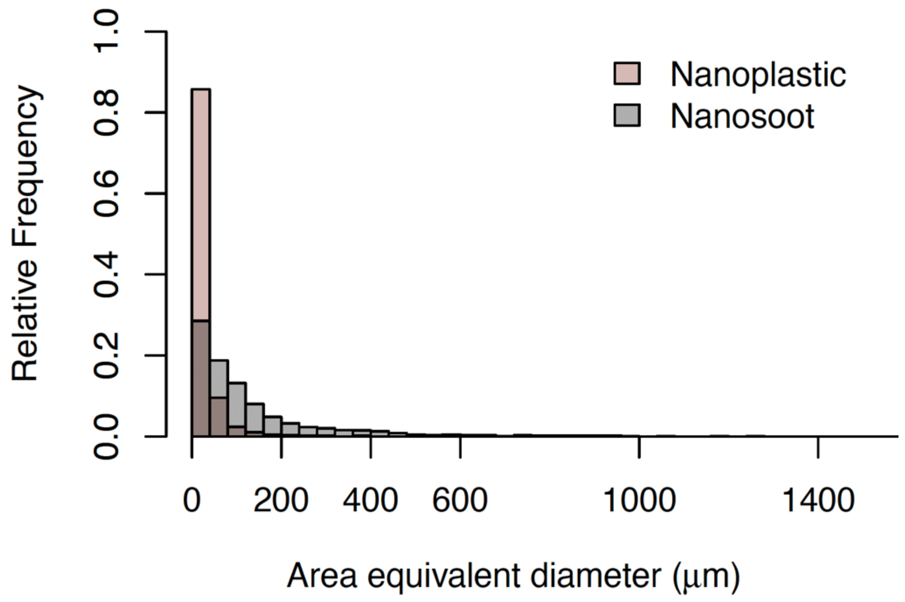

Figure S7: Size distribution of NPs and nanosoot aggregates recovered from thawed ice observed by optical microscopy (n= 1251 for nanosoot, n = 1394 for NPs). Note that while the x-axis starts at 0 mm, the smallest aggregates that were measured were 6.98 and 13.96 mm for NPs and Nanosoot, respectively.

Supplementary Section 2 : Leaching of Yttrium from PP-MP in ASW

4mL syringes were fitted with 0.2 µm regenerated cellulose filters. Each syringe was filled with approximately 12 mg of large PP-MP by removing the pushing element. Control syringes contained no PP-MP. First, to remove any particles that could pass the filter, DI water was added and each syringe and its content was rinsed once. Syringes were reopened and 3mL of ASW was added. After closing the syringes again, they were set to agitate for 24 hours at room temperature. Note that the freezing experiments only lasted 19 hours, so this was a conservative assessment of the maximum possible extent of metal tracer leaching. At the end of each mixing duration, 3 mL of the leachate was pushed through the syringe and diluted to a total volume of 5mL before being analysed by ICP-MS, following the same analysis method as for the plastic digestates. The mass of Yttrium leached per mass of Yttrium contained in the plastic was 0.36 ± 0.12 w/w %.

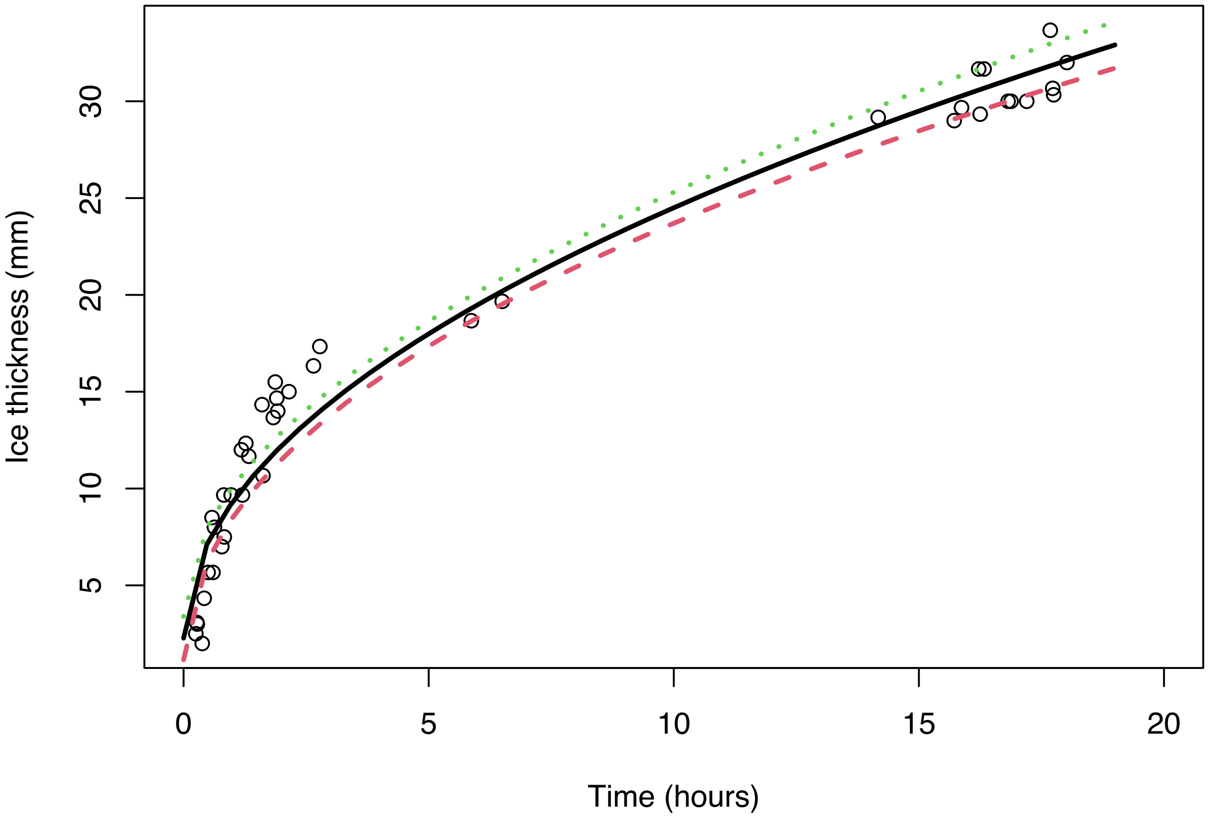

Figure S8: Evolution of the ice thickness (mm) as a function of freezing duration (hours). Each dot respresents an observation point across multiple experimental runs. The modeled growth rate is depicted by the black line, with the green and red dashed lines showing the confidence intervals. Ice thickness = 2.28 mm + 7.03 x $(\text{Time})^{0.5}$ with an adjusted R-squared of 0.9623.

Supplementary Section 3 : Pre-concentration of NPs by flocculation.

NPs were pre-concentrated prior to digestion by flocculation. Centrifugation tubes with a capacity of 50 mL were filled with a maximum volume of 40 mL of each sample, and the mass of each sample was measured. After settling overnight, a polyacrylamide co-acrylic acid (PAM) solution (100 g L^{-1}, pH 9, Sigma-Aldrich) and aluminum nitrate nonahydrate solution (67.093 g L^{-1} of $Al(NO_3)_3$ $9H_2O$) were added to achieve concentrations of 2 800 mg L^{-1} PAM and 1 400 mg L^{-1} Al in each tube. The samples were mixed for 1.5 hours with an end-over-end shaker (Level 7 out of 10), allowed to settle overnight and then centrifuged at 10 000 g for 2 hours using an Allegra X-30R centrifuge (Beckman Coutler). After centrifugation, the supernatant was removed and the flocs and the residual 200 µL at the bottom of the tube were retained for further analysis. To transfer the flocs into the microwave digestion tubes, each centrifugation tube was rinsed with 0.575 mL of distilled HNO_3 at 65% (puriss. Sigma-Aldrich). This rinsing procedure was repeated a total of four times to ensure a complete and quantitative transfer. Recovery tests were performed with ASW with the same salinity and NOM content as the underlying liquid, the ice and the brine and showed an overall recovery rate of 95 ± 5 %.

Table S3: Mass balance of species from the columns after experiments (%, Average ± Standard deviation. n= 42 for water and Sea salts, n = 5 for Rose Bengal, n = 4 for Nanosoot, n= 6 for NP, PET-MP Small, PP-MP Large and Small, n= 9 for PET-MP Large)

Water	Salts	Rose Bengal	Nanosoot	NP	Small PET	Large PET	Small PP	Large PP
99.55 ± 1.04	101.97 ± 4.94	96.95 ± 0.57	96.35 ± 3.32	95.75 ± 2.35	98.68 ± 6.17	89.86 ± 3.81	73.60 ± 20.38	88.49 ± 35.28

Table S4: p-values of Welch t-test between control and contaminants for ice and brine.

	Nanosoot	NP	PET-Large	PET-Small	PP-Large	PP-Small	Rose Bengal
Mass of ice	0.6547	0.523	0.4486	0.3657	0.558	0.4858	0.2334
Mass of brine	**0.002959***	0.4206	0.791	0.182	**<0.001 *****	0.0987	**0.00012 ****
Salinity of ice	0.364	0.1105	0.9468	0.7461	0.9761	0.3092	0.3496
Salinity of brine	0.132	0.4204	0.6442	0.3119	0.4851	0.3119	0.871
pH of ice	0.177	0.06428	0.1938	0.6306	0.07325	0.7806	0.3089
pH of brine	**0.009538 ****	0.2038	**0.01268 ***	0.1607	0.7309	0.1607	**0.001013 ****

Figure S9: Mass, salinity and pH of ice for controls and treatments

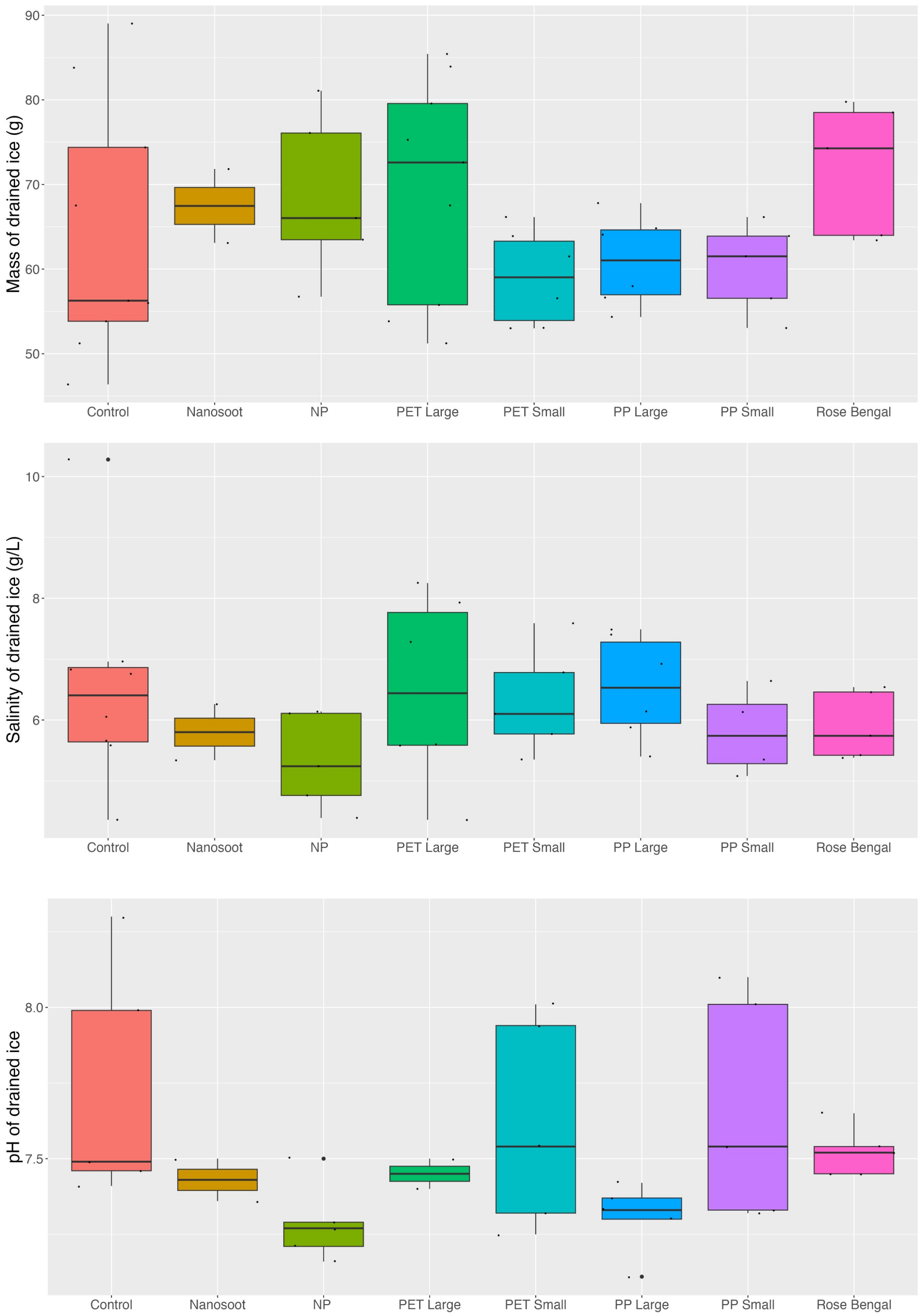

Figure S10: Mass, salinity and pH of brine for controls and treatments

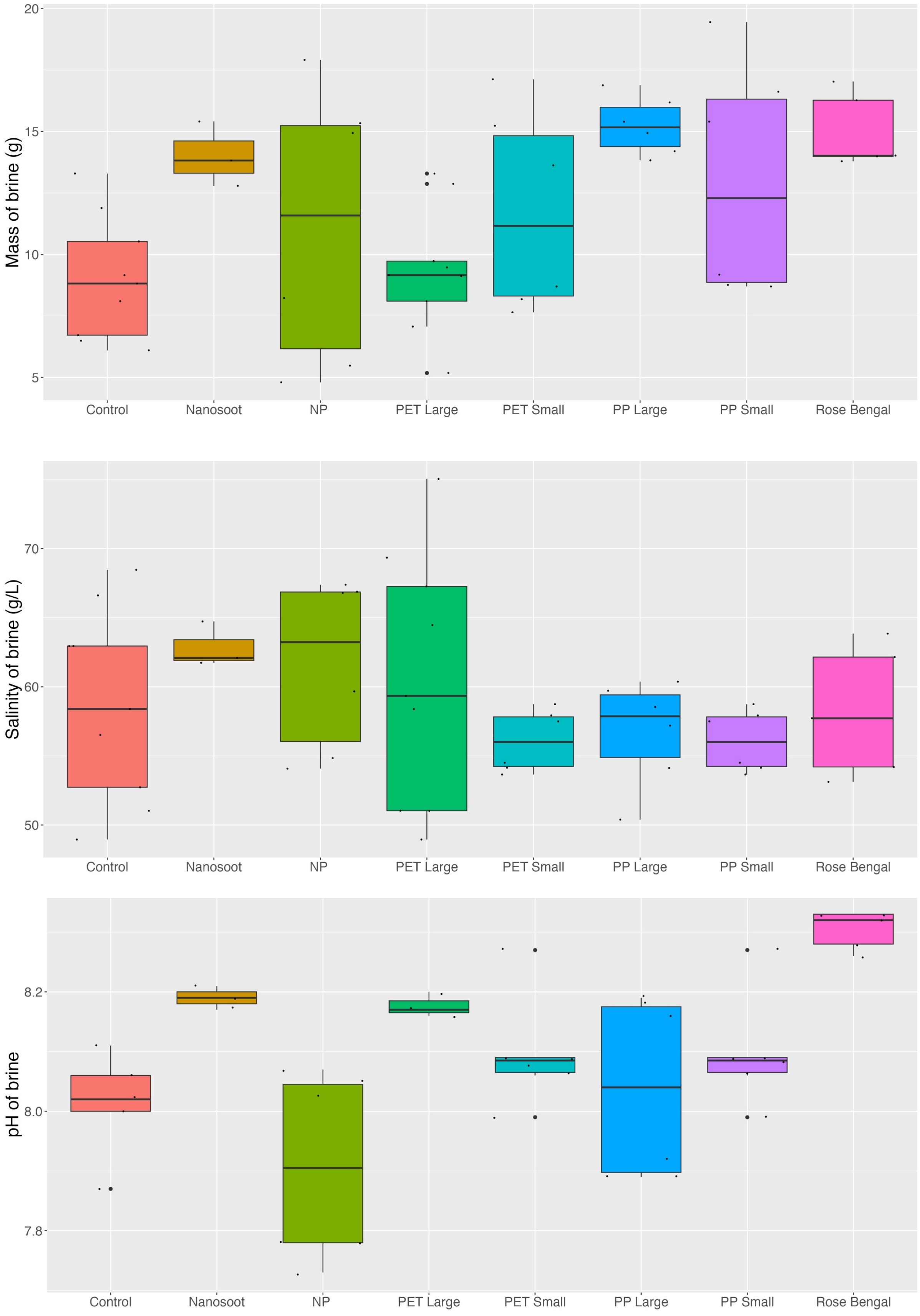

Supplementary References

1. Hare, A. A. *et al.* pH evolution in sea ice grown at an outdoor experimental facility. *Marine Chemistry* **154**, 46–54 (2013).
2. Veselý, L., Štůsek, R., Mikula, O., Yang, X. & Heger, D. Freezing-induced acidification of sea ice brine. *Science of The Total Environment* **946**, 174194 (2024).
3. Parrella, F., Brizzolara, S., Holzner, M. & Mitrano, D. M. Impact of heteroaggregation between microplastics and algae on particle vertical transport. *Nat Water* **2**, 541–552 (2024).
